# Sox2 in the differentiation of cochlear progenitor cells

**DOI:** 10.1038/srep23293

**Published:** 2016-03-18

**Authors:** Judith S. Kempfle, Jack L. Turban, Albert S. B. Edge

**Affiliations:** 1 Department of Otology and Laryngology, Harvard Medical School, Boston, MA 02115, USA; 2 Eaton-Peabody Laboratory, Massachusetts Eye and Ear Infirmary, Boston, MA 02114, USA; 3 Program in Speech and Hearing Bioscience and Technology, Division of Health Science and Technology, Harvard & MIT, Cambridge, MA 02139, USA

## Abstract

HMG domain transcription factor, *Sox2*, is a critical gene for the development of cochlear hair cells, the receptor cells for hearing, but this has been ascribed to expansion of the progenitors that become hair cells. Here, we show that *Sox2* activated *Atoh1*, a transcription factor important for hair cell differentiation, through an interaction with the 3′ enhancer of *Atoh1*. Binding to consensus sequences in the *Atoh1* enhancer was dependent on the level of *Sox2*, and the extent of enhancer binding correlated to the extent of activation. *Atoh1* activation by *Sox2* was required for embryonic hair cell development: deletion of *Sox2* in an inducible mutant, even after progenitor cells were fully established, halted development of hair cells, and silencing also inhibited postnatal differentiation of hair cells induced by inhibition of γ-secretase. *Sox2* is thus required in the cochlea to both expand the progenitor cells and initiate their differentiation to hair cells.

Sox2, an HMG domain transcription factor, plays multiple roles, most prominently in cellular reprogramming and stem cell pluripotency. It accomplishes this by inhibiting transcription of differentiation factors for specific cell types, acting as a transcriptional repressor at a large number of genes and their regulatory regions^1^. The effector activity of Sox2 is exerted through DNA binding and is dependent on level^2^,^3^. It is widely expressed in the embryo and in progenitor cells of various organs as well as the developing and mature nervous system. In the otic placodes from which the ear develops, Sox2 is prominent^4^,^5^, and it is a marker for the prosensory domain in the developing cochlea from which the cochlear and vestibular epithelia develop^5^,^6^. It is extinguished shortly after birth in cochlear hair cells but continues to be expressed in type 2 vestibular hair cells and in supporting cells of vestibular and cochlear epithelium^6^. Evidence from a number of mouse mutants suggested a role for *Sox2* in development of the sensory epithelium of the inner ear^4^,^5^. In particular its expression was required for establishment of the progenitor cells^5^, and, indeed, the lack of hair cells in Sox2 hypomorphic mice was attributed to the failure to establish a prosensory domain^5^. Later studies suggested that Sox2 was antagonistic to bHLH transcription factor, *Atoh1*^4^, similar to what has been shown for proneural transcription factors in other systems^7^,^8^,^9^, but other recent studies showed an upregulation of *Atoh1* by *Sox2*^10^,^11^,^12^,^13^. Sox2 was also shown to be important for auditory neuron differentiation^14^. We sought to determine its overall role in the development of hair cells, the receptor cells for hearing.

Hair cells are present in limited number and although spontaneous regeneration of hair cells has been noted in early postnatal ears^15^,^16^,^17^, this capacity is lost in the adult ear^18^. As a result, loss of hair cells due to environmental toxins, noise, and aging can lead to permanent deafness. A better understanding of mechanisms for the differentiation of hair cells could contribute to new therapies to regenerate hair cells. Supporting cells surround hair cells in the sensory epithelium; they include a subset expressing leucine-rich repeat-containing G protein-coupled receptor 5 (Lgr5) that can act as Wnt-responsive progenitors for hair cells^19^. Notch signaling inhibits bHLH transcription factor, *Atoh1*, a key gene for hair cell development^20^,^21^,^22^,^23^. Notch inhibition increases *Atoh1* expression when Wnt signaling is active^24^. Notch signaling influences cochlear progenitor cell fate by its effect on bHLH transcription factor expression, and the effects of Notch on cochlear progenitors could be mediated by *Sox2*, which is regulated by Notch. We show here that *Sox2* expression is required for the late stages of progenitor cell differentiation to hair cells and stimulates *Atoh1* expression through a concentration dependent molecular interaction with the 3′ enhancer of *Atoh1*.

## Results

### Interaction between Sox2 and the *Atoh1* 3′ Enhancer

**Figure 1:**
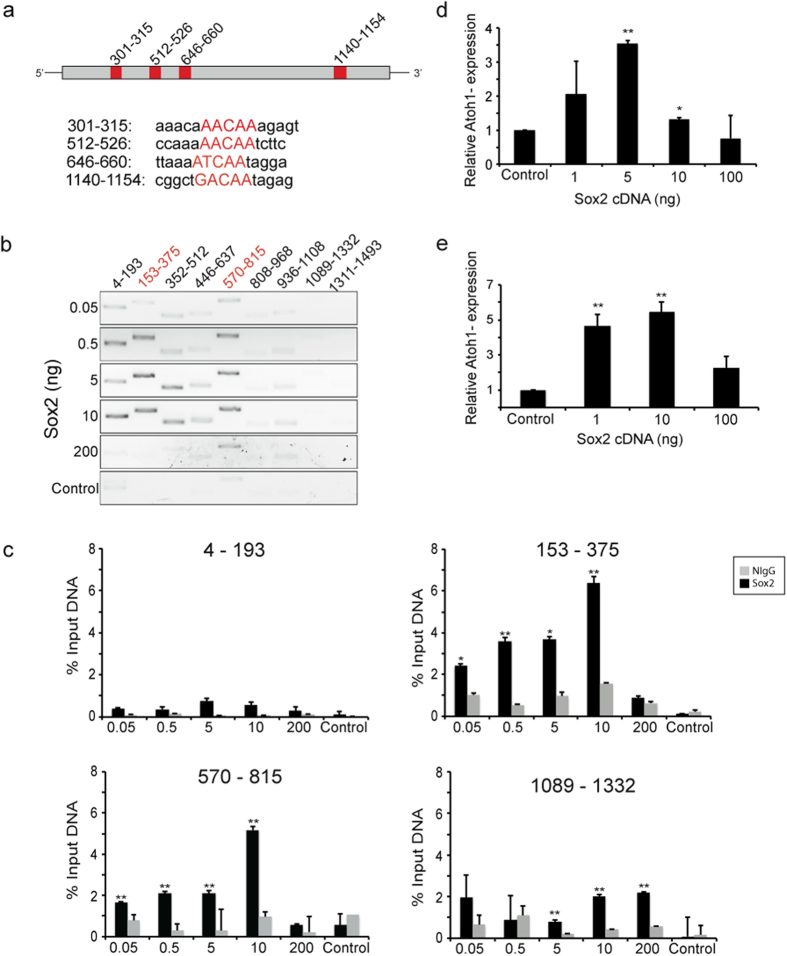
Activation of the *Atoh1* Enhancer by Sox2 Binding. (**a**) The Sox2 binding sequences in the Atoh1 3′- enhancer are shown (red boxes) with the sequences identified by MatInspector (Genomatix). (**b**) ChIP was performed in OC-1 cells after transfection of *Sox2-HA* and the *Atoh1* enhancer. DNA was precipitated with HA antibody (Sox2), followed by RT-PCR with overlapping primer sets covering the entire enhancer. The control represents transfection with empty vector. Positive primer sets, 153–375 and 570–815 are shown in red and encompass the binding sites at 301–315 and 646–660, respectively. The bands in adjacent primer sets were ascribed to incomplete shearing of DNA. (**c**) Quantitative ChIP after transfection of OC-1 cells with *Sox2-HA* showed increased binding with Sox2 concentration up to 5–10 ng/40,000 cells and decreased binding at the higher concentration. Binding at 153–375 and 570–815 sites showed a monotonic increase with Sox2 concentration. The low signal at 4–193 was thought to represent overlap with the site amplified by 153–375. As in (**b**), concentration-dependent binding was not observed at 1089–1332. The control represents transfection with empty vector. ChIP for Sox2 was compared to ChIP for NIgG (*P < 0.05; **P < 0.01). (**d**) Increasing concentrations of *Sox2* cDNA led to maximum upregulation of *Atoh1* at 5 ng, as measured by quantitative RT-PCR in OC-1 cells. (**e**) Upregulation of *Atoh1* was greatest at 10 ng of *Sox2* cDNA, as measured by quantitative RT-PCR in differentiating neurospheres.

*Sox2* is necessary for normal cochlear development, because of its function in the establishment of the prosensory epithelium^5^,^25^,^26^. We and others have shown that Sox2 upregulates bHLH transcription factors, *Atoh1* and *Neurog1*, during determination of cochlear progenitor cell fate^10^,^12^,^27^, as it does in neural progenitor and Merkel cells^11^,^13^,^28^. We found that the level of Sox2 was an important determinant for progenitor cell differentiation or proliferation^27^, as it is for the determination of ES cell fate^29^. To examine the role of Sox2 in *Atoh1* regulation, we assessed Sox2 binding to the *Atoh1* enhancer. Sox2 is an HMG domain transcription factor; its interaction with consensus binding sequences has been characterized^30^. We attempted to determine whether DNA binding to Sox2 was level-dependent and whether alterations in binding of Sox2 to the *Atoh1* enhancer at increasing concentrations correlated to changes in *Atoh1* expression. We assessed binding to Sox2 sites (Fig. 1a shows sites at 301–315, 512–526, 646–660, and 1140–1154 in the *Atoh1* enhancer) by chromatin immunoprecipitation (ChIP) in OC-1 cells, an inner ear cell line that endogenously expresses *Atoh1*^31^. Precipitation of the DNA with an HA antibody after transfection of OC-1 cells with *Sox2-HA* indicated active binding sites in the *Atoh1* enhancer. These sites could be detected by PCR using 9 consecutive primer sets to span the entire enhancer (Fig. 1b; Table S1). The ChIP indicated 2 active binding sites at 301–315 and 646–660 (contained in the primer sets shown in red in Fig. 1a), and usage of the sites was sensitive to the concentration of Sox2. Quantitative ChIP at these sites showed that the strength of binding changed with the level of Sox2. At 301–315, where the highest level of binding was seen, a plateau of DNA binding was reached at 10 ng (Fig. 1c). At this site, higher concentrations decreased the percent of input recovered in the ChIP. Low signals or signals that did not increase monotonically with Sox2 concentration were attributed to incomplete shearing of DNA and these sequences, which included areas with (1089–1332) and without (4–193) canonical sites (Fig. 1c), were assumed to lack active binding sites. Binding to the site at 1089–1332, however, could not be ruled out by the results of the ChIP analysis.

Further testing of Sox2 binding to the *Atoh1* enhancer was performed in HEK cells, and binding was compared to OC-1 cells to determine whether the result was generalizable outside the cochlea. Whereas the overall pattern was similar, differences in the distribution of Sox2 suggested that tissue-specific expression of DNA-binding components or protein interacting partners may fine-tune the binding to specific sites (Fig. S1a). Additional quantitative ChIP was carried out through the use of a construct with a 4X repeat of the Sox2 binding site at 301–315. Sox2 binding to this construct was also dose-dependent up to a maximum, with a decrease at higher concentrations (Fig. S1b).

Atoh1 mRNA levels were measured *in vitro* in OC-1 cells and inner ear neurospheres by quantitative RT-PCR to assess the effect of Sox2. In OC-1 cells, *Atoh1* expression increased with *Sox2* expression to a maximum and decreased upon elevation of *Sox2* to higher levels (Fig. 1d). The results were similar in inner ear neurospheres (Fig. 1e).

We assessed the activation of the *Atoh1* enhancer and the effect of different levels of Sox2 using a construct that drove *luciferase* with the *Atoh1* enhancer to further understand the role of *Sox2* in its expression. Due to low levels of transfection in OC-1 cells, these experiments were performed in *Atoh1*-expressing intestinal cell line, IEC-6. The level of enhancer activity declined when Sox2 concentrations were increased further after reaching a maximum at 0.5 ng/well i.e. at the high levels, Sox2 had decreasing effects on *Atoh1* (Fig. S1c). Additional experiments conducted with a *luciferase* vector made with the 4X repeat of the Sox2 binding site at position 301–315 demonstrated a similar Sox2 level dependence, although reaching a maximum at lower concentrations (Fig. S1d). Together, these experiments demonstrate a concentration-dependent binding and enhancer activation of *Atoh1* by Sox2 that was seen in both cochlear cells and cell lines derived from other tissues.

### *Sox2* Expression in Embryonic Hair Cell Progenitors

**Figure 2:**
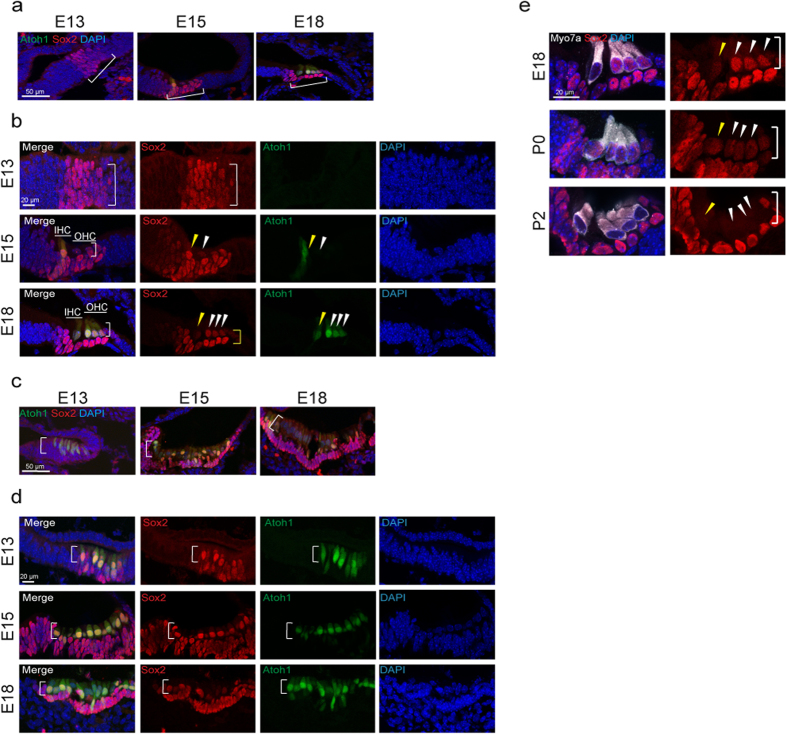
*Sox2* Expression in the Developing Cochlea and Vestibular System. (**a**) Sox2 (red) was expressed in the prosensory cells of the cochlea at E13 (bracket). Sox2 was expressed in the developing sensory epithelium at E15 and E18. (**b**) *Atoh1-nGFP*-positive cells (green) were first found in the sensory epithelium at E15 (bracket and arrowheads). Inner hair cells (IHC, yellow arrowhead), which develop before outer hair cells (OHC, white arrowheads), expressed *Sox2* when newly formed at E15, and the newly formed inner and outer hair cells continued to express *Sox2* at E18. *Sox2* expression in hair cells had begun to decrease at that time (arrowheads) but was strong in supporting cells (yellow bracket). The mid-basal region is shown. (**c**) *Atoh1-nGFP* positive cells could be seen in the developing vestibular system as early as E13 (white bracket) and the cells expressed *Sox2*. (**d**) *Atoh1-nGFP* positive cells continued to express Sox2 at E15 (white bracket). Atoh1-nGFP-positive hair cells showed reduced *Sox2* expression at E18 (white bracket). (**e**) Cochlear hair cells expressed myosin VIIa (Myo7a, white) at E18, P0 and P2. *Sox2* was still expressed in hair cells in the cochlea until P0, but was undetectable in these cells at P2. It continued to be expressed in supporting cells at P2 (bracket and arrowheads).

We next analyzed the expression of *Sox2* during development of the mouse cochlea. Expression of *Sox2* was observed in the prosensory cells of the cochlea at E13 (Fig. 2a,b), and initiation of *Atoh1* expression was observed between E13 and E15 (Fig. 2b).

The area of the developing otocyst marked by *Sox2* expression corresponds to the prosensory epithelium (Fig. 2b). At E15, expression of *Sox2* was apparent in the ventromedial region (Fig. 2b) and was accompanied by GFP expression from the *Atoh1* enhancer. These newly differentiating hair cells that co-expressed Sox2 and Atoh1 can be seen in the upper layer of the sensory epithelium (Fig. 2b). Sox2 expression was seen in cells destined to become both hair cells and supporting cells. Developing hair cells, identified by their expression of Atoh1 as well as myosin VIIa, still expressed Sox2 at E18 (Fig. 2b,e) and loss of Sox2 in hair cells in a gradient from base to apex occurred between P0 (Fig. 2e, P0) and P2 (Fig. 2e, P2).

Sox2 was broadly expressed in the developing vestibular epithelia at E13 (Fig. 2c). Atoh1-expressing cells could be detected at E13 in the vestibular organs, which differentiate earlier than the cochlea (Fig. 2c,d). Co-expression of Sox2 continued in the newly developing, Atoh1-positive, cochlear hair cells (Fig. 2d). Hair cells at E18 expressed myosin VIIa and Sox2 and had not completely lost Sox2 expression in the early postnatal period (Fig. S2). The results in both balance and hearing organs of the developing inner ear thus suggest that expression of Sox2 is maintained throughout the differentiation of hair cells, before becoming undetectable at P2 in cochlear hair cells.

### *Sox2* is Necessary for Hair Cell Differentiation

**Figure 3:**
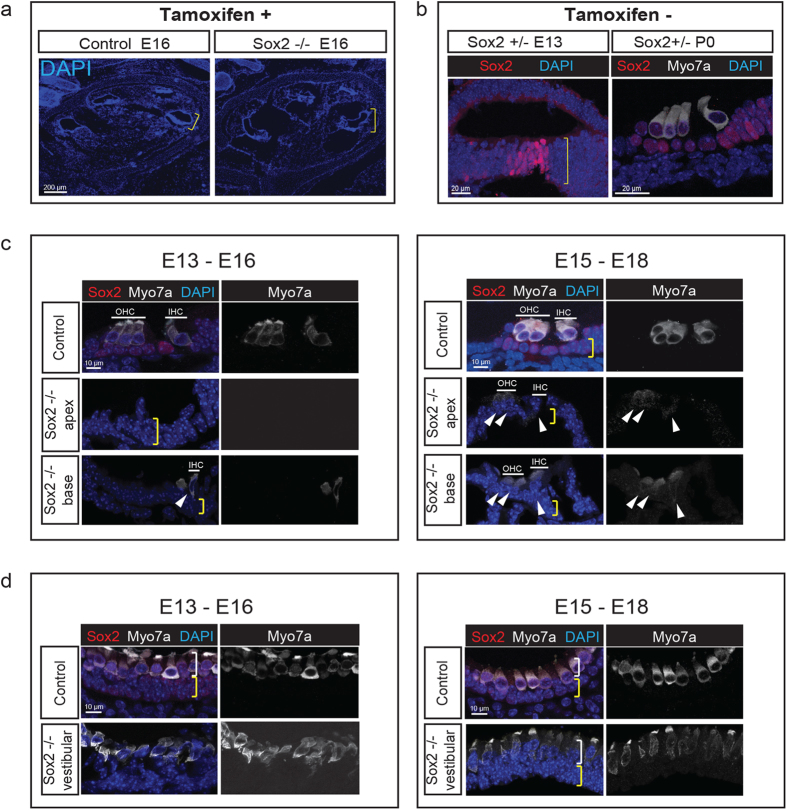
*Sox2* is Required for Differentiation of Cochlear and Vestibular Hair Cells. (**a**) The structure of the cochlea was intact when Sox2 was deleted by tamoxifen administration at E13 and analyzed at E16 (a Cre-negative *Sox2*^*flox/flox*^ control was compared to a *Sox2-Cre-ER; Sox2*^*flox/*^+ knockout, Sox2 −/−). (**b**) The prosensory progenitors were present at E13, and hair cells had formed normally at P0 in an undeleted *Sox2-Cre-ER; Sox2*^*flox/*^+ mouse (Sox2 +/−). (**c**) After deletion of *Sox2* at E13, the cochlea showed a nearly complete absence of hair cells at E16 (no hair cells were seen in the mid and apical regions and one row of dysmorphic inner hair cells was observed at the base). The position of myosin VIIa-positive (white) inner and outer hair cells are indicated by lines labeled IHC and OHC, and the position of the supporting cell layer is indicated by the yellow bracket (note the absence of Sox2 in the tamoxifen-treated animal). One inner and two outer hair cell rows at the base and a small number of dysmorphic hair cells in the apical region (arrowheads) were apparent at E18 when *Sox2* was deleted 2 days later at E15. (**d**) After deletion of *Sox2* at E13, the vestibular organs had weakly myosin VIIa-positive (white), dysmorphic hair cells at E16 in a layer (white bracket) above the supporting cell layer (yellow bracket; compare to the Sox2-positive (red) supporting cells and myosin VIIa/Sox2 double-positive hair cells in control). A greater number of hair cells were observed (white bracket) at E18 after deletion of Sox2 at E15. Nuclei were stained with DAPI (blue).

Sensory progenitor cells in the developing sensory epithelium are established by E13^32^. After tamoxifen administration to delete the floxed *Sox2* gene at E13, we found an overall normal gross morphology at E16 (Fig. 3a). To test whether progenitor cells were present in *Sox2-Cre-ER; Sox2*^*flox/*^+ mice at this time point, we assessed expression by immunolabeling for Sox2. The prosensory domain contained the normal complement of cells in both surface views (Fig. S3) and sections (Fig. 3b, E13) and generated hair cells normally (Fig. 3b, P0) in controls that were not treated with tamoxifen. Examination of the cochlea at E16 after administration of tamoxifen at E13, showed that hair cells had differentiated partially at the base (where hair cell differentiation occurs first), but that deletion of *Sox2* had halted the further differentiation of hair cells. No hair cells were found in the middle and apical regions (Fig. 3c). Few hair cells developed when the gene was deleted at E15 and examined at E18 (Fig. 3c).

Deletion of *Sox2* at E13 (Fig. 3d) did not affect initial appearance of hair cells in the vestibular sensory epithelia, consistent with earlier development of hair cells in these organs, although the hair cells were short and irregularly shaped, with low myosin VIIa expression, and pyknotic nuclei. Again, further development was seen when the deletion was performed at E15. The abrogation of hair cell differentiation by deletion of *Sox2* in ears after the formation of sensory progenitor cells indicates that Sox2 is necessary not only for establishing the sensory progenitors^5^, but for the differentiation of hair cells from the sensory progenitors.

### Expression of *Sox2* in Progenitor Cells Differentiating to Hair Cells

**Figure 4:**
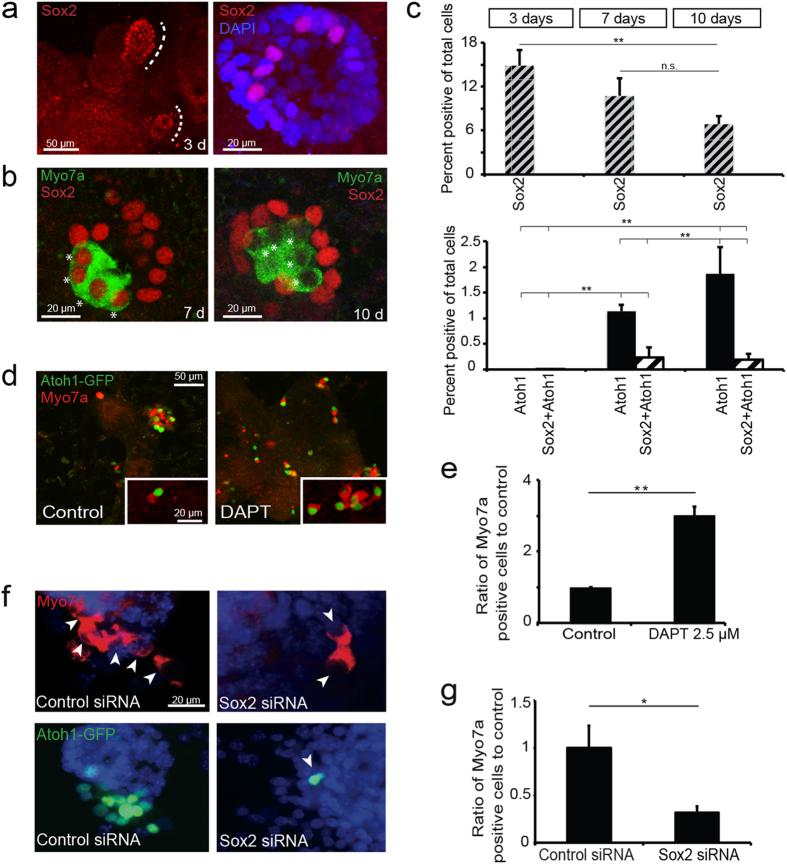
Requirement of Sox2 for the Formation of New Hair Cells from Inner Ear Spheres. (**a**) Distinct patches of Sox2-positive cells were seen in differentiating spheres at 3 days (dotted line). (**b**) In progenitors differentiated for 7 days, all myosin VIIa-positive cells (green) co-expressed *Sox2* (asterisks). Many myosin VIIa-positive cells had lost Sox2 expression at 10 days. (**c**) Quantification of Sox2-positive cells (top graph) at 3,7 and 10 days of differentiation (**P < 0.01). Bottom graph shows quantification of Atoh1-positive cells (black columns) and the fraction of Atoh1-Sox2 double positive cells (patterned columns). Atoh1-positive hair cells and Sox2- Atoh1 double-positive hair cells were not seen during early differentiation and increased significantly at later time points (**P < 0.01). (**d**) The number of newly formed hair cells after treatment with DAPT increased and hair cells were Atoh1 (green) and myosin VIIa (red)-positive (insert). (**e**) DAPT treatment increased the proportion of myosin VIIa-positive cells compared to control (**P < 0.01). (**f**) Neurospheres under differentiating conditions in the presence of DAPT and treated with siRNA for *Sox2* (100 nM total) had a reduced number of myosin VIIa (top panel) or Atoh1-nGFP (bottom panel) positive cells (arrows) compared to control siRNA after differentiation for 7 days. The transfection efficiency was 40–50%. (**g**) The reduction in the number of hair cells was significant (*P < 0.05).

*Sox2* is expressed in inner ear progenitor cells^33^ and, even though the cultures are heterogeneous, we have previously demonstrated an overall decrease in progenitor markers and an increase in hair cell markers when inner ear progenitors are transferred from a proliferative self-renewing culture (as floating neurospheres in growth factors) to a differentiating culture (as adherent cells in the absence of growth factors). *Atoh1* activation was followed by *GFP* expression in spheres from *Atoh1-nGFP* mice. At 3 days of differentiation, groups of Sox2-positive cells were observed in the cultures (Fig. 4a,c). After 7 days in culture, immature hair cells (myosin VIIa and Atoh1 staining cells) differentiated and showed strong staining for Sox2 (Fig. 4b,c). The total number of hair cells (Atoh1-GFP positive cells) increased during hair cell differentiation between day 7 and 10, whereas the percentage of immature hair cells (Sox2 and Atoh1 double-positive) decreased upon maturation (Fig. 4c).

### Effect of Sox2 on Differentiation of Hair Cells from Inner Ear Progenitors

Inhibition of Notch signaling increases differentiation of hair cells from inner ear progenitor cells isolated as neurospheres^27^ by altering the expression of *Atoh1*. It may exert this influence through an effect on *Sox2*, in addition to the effects mediated by Notch effectors, Hes and Hey^12^. The level of Sox2 was decreased by 30% in cells treated with γ-secretase inhibitor, DAPT (Fig. S4a), but the treatment did not alter the number of Sox2-positive cells (Fig. S4b), indicating a reduction of Sox2 levels in preexisting progenitors upon differentiation into hair cells. Treatment with DAPT increased the percentage of Atoh1-single-positive and Atoh1-Sox2-double-positive cells obtained from inner ear progenitor cells almost 4-fold (Fig. S4c).

A significant increase in myosin VIIa-positive cells was apparent after treatment of inner ear progenitor cells with DAPT for 7 days (Fig. 4d,e). To evaluate whether hair cell differentiation induced by a γ-secretase inhibitor depended on expression of *Sox2*, we silenced *Sox2* with siRNA. Effective reductions in *Sox2* RNA levels (70%; Fig. S4d) were observed in inner ear progenitor cells. *Sox2* silencing with siRNA decreased the number of DAPT-induced Atoh1- and myosin VIIa-positive cells (Fig. 4f), and the decrease was significant (Fig. 4g). Thus, *Sox2* expression at the correct level was important for both the upregulation of *Atoh1* and hair cell differentiation that resulted from γ-secretase inhibition.

### Sox2 in Hair Cell Differentiation

**Figure 5:**
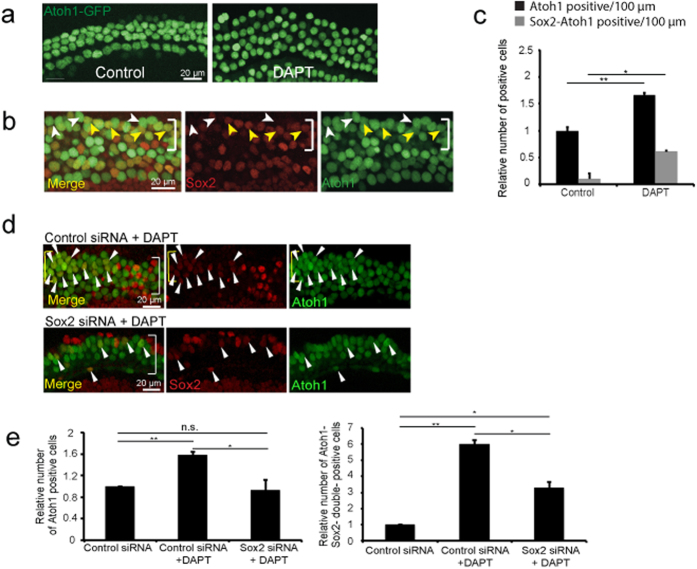
Requirement of *Sox2* for Hair Cell Differentiation in γ-Secretase-Treated Organ of Corti. (**a**) Organ of Corti explants from neonatal *Atoh1-nGFP* mice (P1-P2) were kept in culture for 48 hours and treated with DAPT (2.5 μM) or carrier (DMSO, 0.1%) as a control. An increased number of hair cells was seen (mid-apical region is shown). (**b**) At 48 hours of DAPT treatment, Sox2-positive supporting cells that upregulated *Atoh1-nGFP* were observed in the hair cell layer at a position below the original hair cells (bracket). New supporting cell-derived hair cells showed a reduced level of Sox2 compared to supporting cells and were weakly *Atoh1-nGFP*-positive (yellow arrowheads), whereas original hair cells showed stronger *Atoh1-nGFP* expression and no Sox2 expression (white arrowheads). (**c**) Treatment with DAPT reduced Sox2 in supporting cells and increased the percentage of *Atoh1-nGFP* positive cells/100 μm and new *Atoh1-nGFP* positive cells (black columns) that expressed *Sox2* (grey columns) after 72 hours (*P < 0.05). (**d**) Control siRNA (100 nM) with DAPT (2.5 μM) treatment resulted in an increased number of Atoh1-positive cells. Treatment with DAPT (2.5 μM) and *Sox2* siRNA (100 nM total) partly suppressed the increase in new hair cells. White arrowheads mark Sox2-Atoh1 double-positive hair cells. (**e**) *Atoh1-nGFP* positive and Sox2-Atoh1 double-positive outer hair cells (OHC) per 100 μm in the apical region of organ of Corti explants treated with control siRNA, control siRNA and DAPT, or *Sox2* siRNA and DAPT revealed that DAPT treatment significantly increased the number of hair cells in control siRNA-treated explants. *Sox2* siRNA reduced the number of newly formed hair cells (**P < 0.01).

To determine whether *Sox2* levels played a role in accumulation of new hair cells after Notch inhibition in the newborn organ of Corti, Sox2 was followed in explants treated with DAPT, which reduced Sox2 levels in supporting cells^34^,^35^. At 48 hours, additional *Atoh1-nGFP*-positive outer hair cells were seen in the apex (Fig. 5a,c). New *Atoh1-nGFP*-positive hair cells derived from supporting cells expressed Sox2 (Fig. 5b,c). The original hair cells expressed high levels of Atoh1 but no detectable Sox2, while new hair cells expressed a lower level of Sox2 than adjacent supporting cells (Fig. 5d). Expression of Sox2 was lost later during maturation of hair cells. *Sox2* silencing with siRNA decreased the number of both Atoh1-single-positive and Atoh1/Sox2-double-positive cells induced by DAPT (Fig. 5d,e).

## Discussion

We show here that *Sox2* plays an important role in the upregulation of transcription factor, *Atoh1*, in both embryonic and postnatal cochlear progenitor cells, thus triggering the differentiation of hair cells. This is an important auxiliary role of *Sox2*, which also functions to establish prosensory cells in the cochlea, and is indicative of a proposed role for *Sox2* throughout the body, not only in the maintenance of stem/progenitor cells, but in the specification of mature cell fate. However, this is a role for *Sox2* that is seemingly at odds with its inhibition of differentiation factor expression. Since *Atoh1* is a key gene for specifying hair cell fate, upregulation of *Atoh1* expression by Sox2 binding to DNA in the regulatory region is a key upstream event in differentiation.

*Sox2* inhibits the expression of differentiation factors^1^,^7^,^8^,^9^, and functions as a core factor for stem cell pluripotency along with Oct4 and Nanog^1^. Sox2 is, in fact, one of four transcription factors that reprogram various cell types to induced pluripotent stem cells^36^. Sox2 plays a role in the development of neural progenitors in the CNS^37^ and acts to inhibit the expression of neural transcription factors. *Sox2* downregulation is thought to be an essential step for progenitors to undergo commitment to neuronal differentiation via expression of proneural genes^7^,^29^,^38^, and a similar mechanism, in which *Sox2* had to be downregulated^4^,^5^ to permit hair cell genesis was proposed for the inner ear^4^.

Our data suggest a role for *Sox2* in the activation of proneural transcription factors and, thus, in cell fate determination. Deletion of *Sox2*, after the prosensory cells had generated the prosensory domain, but before they had become hair cells, prevented hair cell differentiation. Such a role for *Sox2* would suggest that it both maintained progenitor cell potency and initiated differentiation. This apparent paradox may be resolved by the timing of expression, i.e. the function of *Sox2* would be, first, to establish the progenitor cells and, subsequently, to specify cell fate by a positive effect on a differentiation factor. The switch in roles could be due to an altered level of Sox2 or to the changing status of the progenitor cells during the course of inner ear development. *Sox2* expression comes up at the earliest stages of cochlear development, when the otic placode first appears, and during the formation of the otocyst and the prosensory domain, for which its expression is necessary^5^. These progenitor cells are the source of both sensory cells and neurons, both of which require *Sox2* expression for normal development^5^,^14^. We traced the expression of Sox2 throughout the formation of hair cells and found that it was expressed not only in hair cell progenitors, but also in newly born hair cells. Its expression diminished in a gradient from base to apex, and it was permanently downregulated in hair cells of the cochlea soon after birth. The importance of *Sox2* for bHLH transcription factor expression has also been observed in neurons, in which *Sox2* expression upregulates the expression of *Neurog1*^27^, and in Xenopus, where *Sox2* was implicated in the conversion from proliferation to differentiation of neural progenitors^28^. Subsequent to differentiation, *Sox2* appeared to be inhibited by downstream transcription factors in a feedback loop that prevented excess neurogenesis^7^,^9^. Interaction of Sox2 with other transcription factors is another potential mechanism allowing Sox2 to play different roles in development^10^,^39^.

The role of *Sox2* as a differentiation factor is at odds with a global antagonism of proneural transcription factor activity, but is not inconsistent with an eventual antagonistic role. We found that a reduction in Sox2 level was necessary for proneural transcription factors to be expressed *in vitro* and correlated with hair cell differentiation from supporting cells brought about by treatment of cochlear explants *in vitro* with DAPT. We think that this reduction is necessary for an active role of *Sox2* in differentiation, not simply to release an inhibitory influence. Decreased *Atoh1* expression at higher levels of Sox2 could be due to insufficient levels of the DNA-binding protein complex or to competition with other DNA-binding factors^1^. This criticality of concentration for Sox2 is consistent with the CNS^40^, where gene dosage leads to alternate activities of *Sox2*^4^,^41^, including cell differentiation. The level of Sox2 in hair cells subsides further and is eventually extinguished after hair cell differentiation.

*Sox2* is downstream of Notch and its expression is decreased by treatment with a γ-secretase inhibitor^27^,^42^. Notch signaling is necessary for the early stages of cochlear sensory development and may act in part by upregulating *Sox2* in progenitor cells, whereas loss of Notch signaling due to lateral inhibition could drop Sox2 levels in a progenitor cell that becomes a hair cell. We show here that *Sox2* is necessary for the effects of Notch inhibition on hair cell differentiation in newborn explants and cochlear spheres. The antagonism between Atoh1 and Sox2 may be attributable to an effect of Sox2 on Notch effectors such as Hes1 and Hes5 that antagonize bHLH transcription factor activity^12^. However, effects of Notch on proliferation were independent of Sox2^42^.

Heterozygous mutations in *Sox2* in humans result in eye defects that can be accompanied by deafness^43^. *Sox2* knockout animals have severe deficiencies in the development of the inner ear, but, because *Sox2* is expressed early in development of the mouse inner ear - well before *Atoh1* - and because its loss resulted in incomplete initial formation of the sensory epithelium^25^,^26^,^44^,^45^, it was difficult to assess any role in hair cell differentiation prior to this work. The current results with conditional knockout show that *Sox2* plays a critical role in the differentiation of hair cells from prosensory epithelial progenitor cells.

## Methods

### Chromatin Immunoprecipitation (ChIP)

Consensus sequences for Sox2 binding in the *Atoh1* 3′-enhancer were initially identified using the MatInspector software tool for transcription factor binding sites (Genomatix). OC-1 and 293T (HEK, Thermo Fisher) cells were transfected at 30% confluency using Lipofectamine (3 μl/μg DNA/ml) and Sox2-HA cDNA (0.01–200 ng/40,000 cells) in Opti-MEM. After 48 hours, cells were harvested and processed for ChIP (Active Motif, ChIP-IT Express Enzymatic). Chromatin was precipitated with mouse anti-HA antibody (3 μg), or normal rabbit IgG (3 μg).

### Quantitative RT-PCR

Total RNA was extracted with the RNeasy Maxi Kit (Qiagen) according to the manufacturer’s instructions from embryonic tissue harvested in RNAlater (Ambion) or from OC-1 cells or spheres transfected using Lipofectamine with different concentrations of Sox2 cDNA (in ng/40,000 cells) for 48 hours or treated with DAPT or DAPT and siRNA. RNA was denatured at 65 °C for 5 min. For reverse transcription, ImProm II (Promega) was used with random hexamers. The reverse transcription conditions were 25 °C for 5 min followed by 42 °C for 60 min. The reaction was terminated at 70 °C for 15 min. The cDNAs were mixed with Platinum quantitative PCR Supermix ROX with UDG (Invitrogen) and primers for 18S RNA, Atoh1 or Sox2 (ABI) according to the manufacturer’s instructions (primers are listed in Table S1). Gene expression was measured relative to 18S RNA. Samples were analyzed in 96 well plates in triplicate by quantitative PCR (Applied Biosystems 7900HT) using the following conditions: initial denaturation at 95 °C for 2 min, denaturation at 95 °C for 40 s and annealing/extension at 60 °C for 35 s for 45 cycles. All statistical analysis was performed with the Student’s t-test.

### Plasmids

A vector containing a 1.4 kb enhancer for *Atoh1*, sufficient for endogenous *Atoh1* expression^46^, cloned upstream of *luciferase*^27^, was used in luciferase assays. *Sox2-HA* (pCAG-HA-Sox2-IP) was from Addgene. A 4x repeat of a 41 base pair enhancer sequence (acccaaacaaacaaagagtcagcacttcttaaagtaatgaa) spanning the binding site at position 301–315 was synthesized (GenScript) and cloned into pGL4 (Promega).

### Luciferase Assay

IEC6 cells (ATCC) were seeded onto 96-well plates 1 day before transfection. IEC6 cells are an intestinal cell line that shows high levels of Atoh1 and increased responsiveness to induction. At 40–50% confluency, 100 ng of luciferase reporter construct (intact Atoh1 enhancer or 4x-binding site reporter), 10 ng of Renilla luciferase construct and different amounts of Sox2 effector (from 0.1 ng–100 ng) were transfected with Lipofectamine 3000 (3 μl/μg DNA/ml, Life Technologies) into the cells for 6 h. Cells were lysed after 48 h, and luciferase activity was measured using the Dual Luciferase Reporter Assay System (Promega) according to the manufacturer’s instructions in a Wallac Victor^2^ 1420 Multilabel Counter (Perkin-Elmer).

### Isolation of Organ of Corti Cells for Neurosphere Culture

For each experiment, cochleae of 4–6 neonatal C57BL/6 or *Atoh1-nGFP* pups^47^ that express *GFP* under the control of the *Atoh1* enhancer (a generous gift from Jane E. Johnson, University of Texas) were dissected in HBSS and the organ of Corti was separated from the stria vascularis and the spiral ganglion neurons. The tissues were dissociated in trypsin (0.05%) for 13 min in PBS at 37 °C. 10% FBS in DMEM-high glucose medium was used to stop the reaction. After washing, the tissue was manually dissociated. The triturated cells were then passed through a 70 μm cell strainer (BD Labware) to remove tissue debris. Single cells were cultured in DMEM/F12 (1:1) supplemented with N2 and B27 (Invitrogen), and EGF (20 ng/ml; Chemicon), bFGF (10 ng/ml; Chemicon), IGF-1 (50 ng/ml;Chemicon), and heparan sulfate (50 ng/ml; Sigma). Single cells were maintained in ultra-low cluster plates (Costar) for several days in culture to obtain neurospheres. For passage, neurospheres of the first generation were dissociated with a 27G needle and syringe (BD Labware) 6–8 times. Single cell suspensions were cultured in fresh medium F12/DMEM (1:1) with the same growth factors to form neurospheres until use at the 4^th^ to 5^th^ generation.

### Immunohistochemistry of Neurospheres

Differentiated neurospheres were fixed at room temperature in 4% paraformaldehyde/PBS for 10 min and then washed in PBS. Permeabilization and blocking was performed for spheres or sections with blocking solution (0.3% Triton X-100, 15% heat-inactivated goat or donkey serum in PBS) for 1 h. Diluted primary antibody (0.1% Triton X-100, and 10% heat inactivated goat or donkey serum in PBS) was applied overnight at 4 °C. Secondary antibodies were applied for 2 hours at room temperature. Nuclei were visualized with 4,6-diamidino-2-phenylindole (Vector Laboratories). Staining was analyzed with epifluorescence microscopy (Axioskop2 Mot Axiocam, Zeiss) and confocal microscopy (TCD, Leica).

### Antibodies

The antibodies used were monoclonal mouse antibody against myosin VIIa (used at 1:100; Developmental Studies Hybridoma Bank), polyclonal rabbit antibody against myosin VIIa (used at 1:250; Proteus), polyclonal goat antibody against Sox2 (used at 1:300; Santa Cruz). Secondary antibodies for detection of primary antibodies: Alexa Fluor 488, 568, and 647-conjugated (all used at 1:500; Invitrogen). Mouse monoclonal antibody against HA (Sigma, used for ChIP).

### Mice for *in Vivo* Experiments

*Sox2-Cre-ER*^48^ and *Sox2*^*flox/flox*^ mice^49^ (Jackson Labs, stock numbers 013093 and 017593, respectively) were used for *in vivo* knockout experiments. All animal studies were approved by the Institutional Animal Care and Use Committee of Massachusetts Eye and Ear Infirmary according to National Institutes of Health guidelines. Homozygous *Sox2*^*flox/flox*^ mice were mated with hemizygous *Sox2-Cre-ER* mice. Tamoxifen (100 μl, 50 mg/ml, Sigma) was injected intraperitoneally for 2 consecutive days (E12 and E13, or E14 and E15) to obtain hemizygous *Sox2*-*Cre-ER* animals with deleted *Sox2*^*flox*^. *Sox2-Cre-ER*; *Sox2*^*flox*/+^ mice without exposure to tamoxifen were examined at E13 and P0 to exclude potential effects of the undeleted transgenes. Embryos were genotyped and Cre-negative embryos were used as controls. Immunohistochemistry was performed at E16 and E18 in tamoxifen-treated animals.

### Tissue Preparation of Embryos

Embryos of *Sox2-Cre-ER*; *Sox2*^*flox*/+^ mice were collected at E13, E16 or E18 and *Atoh1-nGFP* mice were collected at E13, E15 and E18 (with identification of a positive plug in the morning counted as E0.5), or postnatally at P0 and P2, and fixed in 4% paraformaldehyde for 4 hours at 4 °C. After dehydration with sucrose (5% and 30%), embryos were embedded in OCT and kept at −80 °C. For immunohistochemistry, tissue was cut (12 μm) and then stained as described for neurospheres. Midbasal regions were imaged in the *in vivo* knockout and explant experiments unless otherwise specified.

### Dissection of Organ of Corti Explants

For each experiment, tissue of neonatal pups with C57BL/6 (Jackson Labs) or Atoh1-nGFP background were dissected on ice in HBSS and the organ of Corti was harvested after removal of spiral ganglion neurons and stria vascularis as previously described^50^.

### Differentiation and Treatment of Neurospheres or Organ of Corti Explants with DAPT

4^th^–5^th^ generation neurospheres or organ of Corti explants (P1-P2) were plated without growth factors in 4-well plates (Greiner) on round 10 mm glass coverslips coated with poly-L-lysine (Cultrex) and attachment took place overnight in 10% FBS/DMEM-high glucose (GIBCO). Attachment was ensured with microscopic inspection and the medium was changed to serum-free DMEM-high glucose/F12 (mixed 1:1, GIBCO) and N2 and B27 (Invitrogen). Neuropheres were differentiated for 3, 7 or 10 days. For treatment, DAPT (CalBiochem) (2.5 μM) or control medium containing only DMSO (0.1%) were applied for 7 days on spheres and 48 hours on organ of Corti explants. Cells were harvested and further analyzed by immunohistochemistry as described for cultured neurospheres. Axiovision 4.3 was used for data acquisition and the number of cells was quantified with Metamorph software. Cell counts were expressed as mean ± standard deviation. An average of 1,000 cells were counted for neurospheres or 100 μm for OC explants. Origin software was used for statistical evaluation.

### SiRNA Silencing

Cochlear neurospheres or organ of Corti explants were transfected with siRNA (100 nM ON-TARGET plus, Smart Pool from Dharmacon for *Sox2*, or non-targeting siRNA), using Gene Silencer (Genlantis) for 24 hours according to the manufacturer’s instructions. Transfection efficiency was monitored using siGlo Red Transfection Indicator (Dharmacon). In addition, 2.5 μM DAPT was added during differentiation culture. After transfection, the cells were washed and cultured in DMEM/F12 with DAPT (1:1) and N2/B27 (spheres and explants) or DMEM/10% FBS and DAPT (IEC6 cells and OC-1 cells). For quantitative PCR experiments after siRNA treatment, cells were differentiated for additional 24 hours after transfection. Organ of Corti explants were kept in culture for an additional 24 hours (48 hours total) and differentiating neurospheres were cultured for up to 6 days after siRNA treatment.

### Statistics

Statistical analysis was performed using an unpaired, two-tailed t-test using Origin software. Data generated with cell lines was repeated at least 3 times, data collected from sphere and explant tissue was repeated at least 4 times. Bonferroni correction was used for comparison of multiple variables. Experimental values obtained for each antibody by ChIP were compared to a NIgG control, and luciferase expression values were compared to sample without effector. Brackets are used to indicate the compared values in all other figures with significance indicated by asterisks.

## Additional Information

**How to cite this article**: Kempfle, J. S. *et al.* Sox2 in the differentiation of cochlear progenitor cells. *Sci. Rep.* **6**, 23293; doi: 10.1038/srep23293 (2016).

## Supplementary Material

Supplementary Figures
