# Supplementary material for: Sox2 in the differentiation of cochlear progenitor cells
Source: Sci Rep. 2016 Mar 18;6:23293. doi: 10.1038/srep23293 (PMC4796895; doi:10.1038/srep23293)

**Supplemental Information**

Sox2 in the differentiation of cochlear progenitor cells

Judith S. Kempfle, Jack L. Turban and Albert S.B. Edge


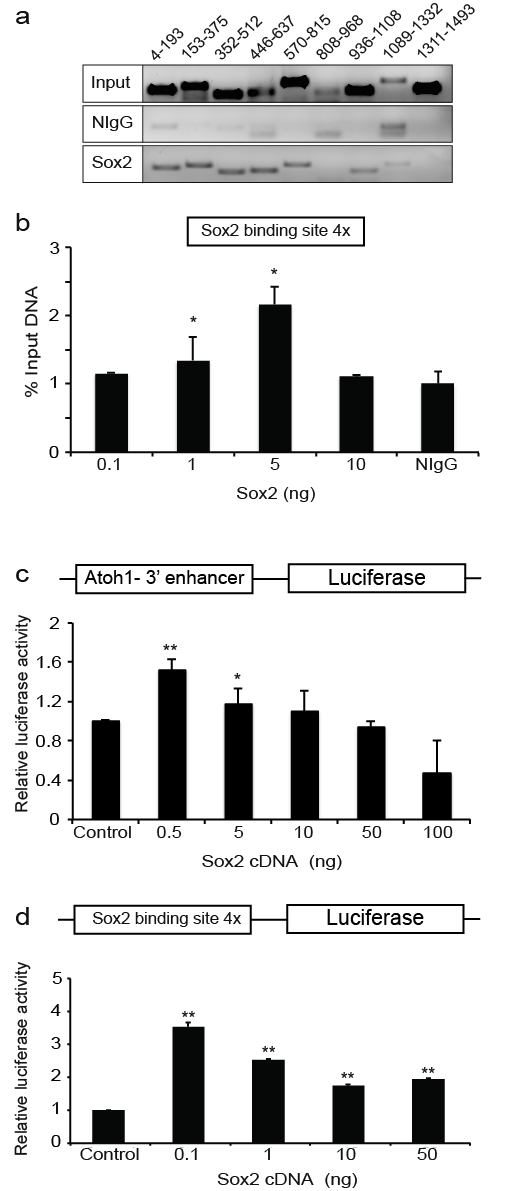


**Figure S1. Sox2 Binds to and Activates the *Atoh1* Enhancer**

(a) ChIP in HEK cells after transfection of *Sox2-HA* and *Atoh1* enhancer. DNA was precipitated with HA antibody (Sox2) or normal IgG (NIgG) as a control followed by RT-PCR with overlapping primer sets covering the entire enhancer. (b) ChIP using a construct containing a 4x repeat of the Sox2 binding site (301-315) demonstrated maximum binding at 5 ng (*Sox2* cDNA concentrations are ng/40,000 cells). Binding was normalized against normal IgG (NIgG) and shown as percentage of input DNA. ChIP for Sox2 and NIgG were compared (*, P < 0.05). (c) Increasing concentrations of *Sox2* cDNA led to maximum upregulation of the enhancer at 0.5 ng as measured by a luciferase assay in IEC6 cells. Higher levels reduced activation of the enhancer. (d) Activation of a 4x repeat of the Sox2- binding site (301-315) in a construct with luciferase demonstrated maximum upregulation at 0.1 ng. Higher concentrations reduced activation. Enhancer transfection served as control in c and d. Transfected concentrations represent cDNA in ng/40,000 cells. Significance was determined relative to control (*, P < 0.05; **, P < 0.01).


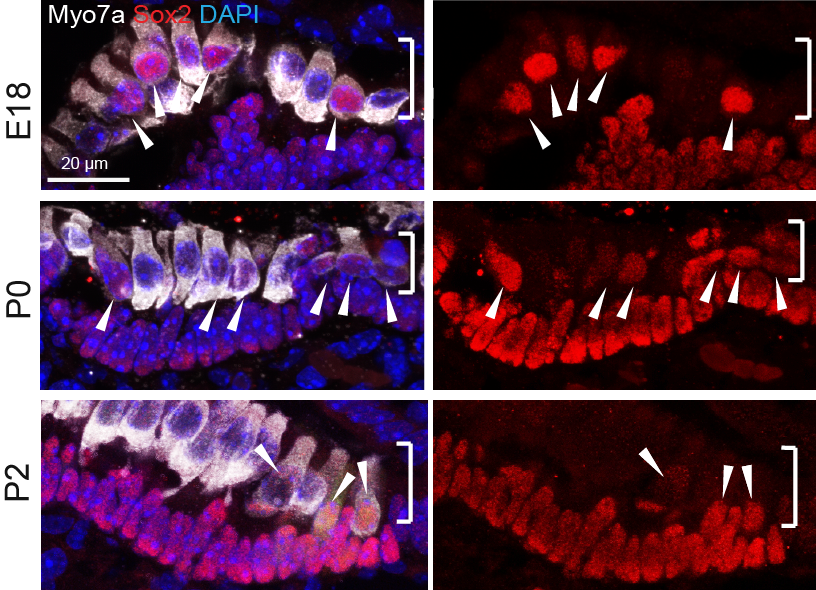


**Figure S2. Assessment of Sox2 Expression in Vestibular Cells**

Vestibular hair cells expressed myosin VIIa (Myo7a, white) at E18, P0 and P2. Expression of Sox2 was maintained (bracket and arrowheads).

**Figure S3. Progenitor Cell Number in Wild Type and Undeleted *Sox2-Cre-ER;Sox2****^flox/+^*

(a) Whole mount preparation of E13 embryonic cochlear duct for the undeleted *Sox2-Cre-ER;Sox2^flox/+^*(Sox2 +/-) and wild type (Control) embryo. No gross changes were observed in the prosensory epithelia. (b) The Sox2-positive cell number was unchanged (n.s., not significant; n=8) in the undeleted ears.

**Figure S4. Effect of DAPT on Atoh1 and Myosin VIIa-Positive Cells in Inner Ear Neurospheres**

(a) Quantitative RT-PCR showed reduced *Sox2* levels after DAPT treatment (**, P < 0.01). (b) Sox2-positive cells were not significantly increased relative to control (DMSO) in DAPT-treated differentiating neurosphere culture (n.s., not significant). (c) Atoh1 positive cells (black) and the proportion of Sox2-Atoh1 positive cells (grey) were increased (**, P < 0.01*, P < 0.05).

(d) *Sox2* siRNA specifically decreased *Sox2* mRNA in differentiating neurospheres (**, P < 0.01).

**Table S1**

Primers used for ChIP of the *Atoh1* 3’-enhancer


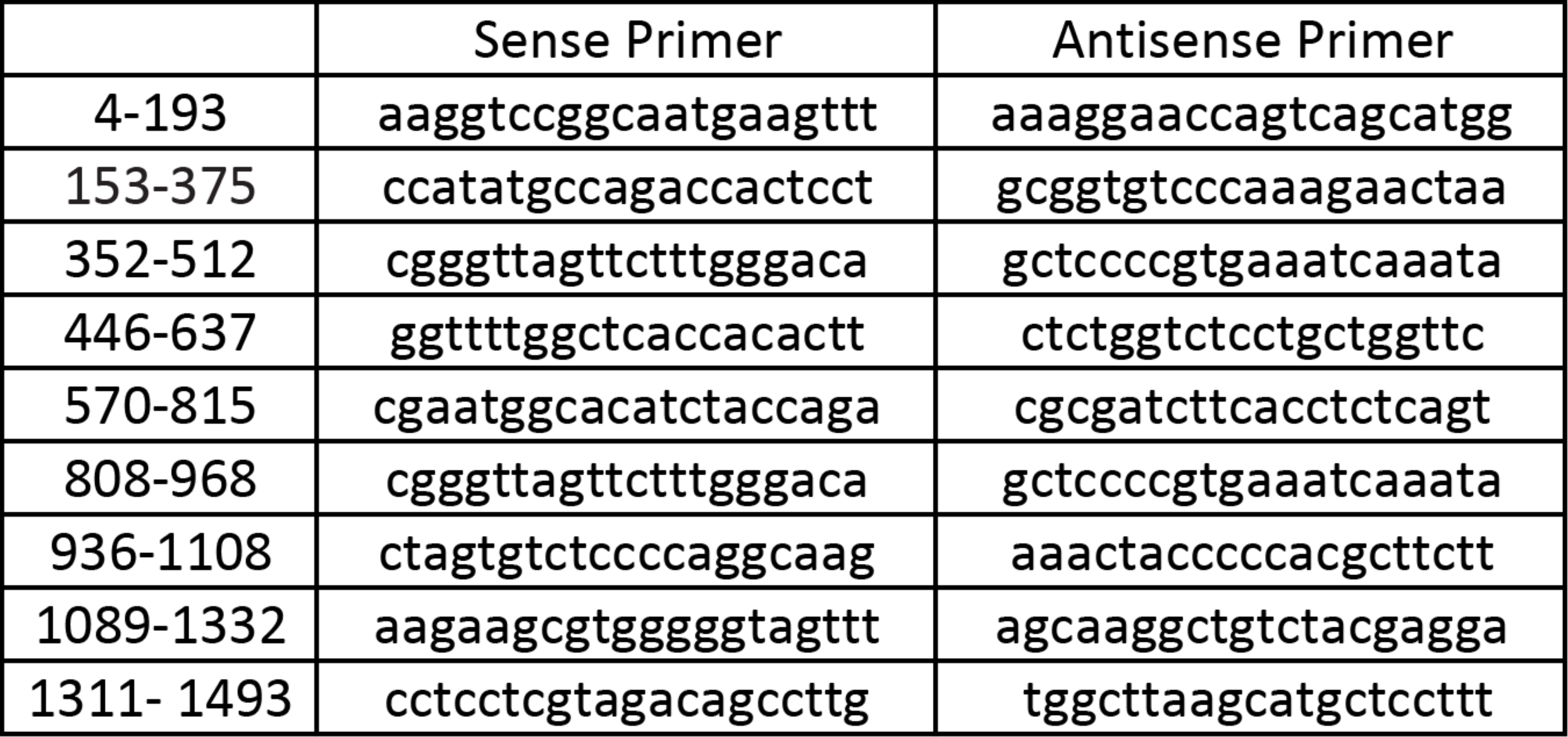

Supplement: Supplementary Figures [file srep23293-s1.doc]
